# Orchestrated Sex: The Representation of Male and Female Musicians in World-Class Symphony Orchestras

**DOI:** 10.3389/fpsyg.2019.01760

**Published:** 2019-08-16

**Authors:** Desmond Charles Sergeant, Evangelos Himonides

**Affiliations:** UCL Institute of Education, University College London, London, United Kingdom

**Keywords:** sex, gender, music performance, classical music, gender representation, symphony orchestra, sex representation, gender equality

## Abstract

This study examines the representation of male and female musicians in world-class symphony orchestras. Personnel of 40 orchestras of three regions, the UK, Europe, and the USA, and distributions of men and women across the four orchestral departments, strings, woodwind, brass, and percussion, are compared. Significant differences in representation between orchestras of the three regions are reported. Practices adopted by orchestras when appointing musicians to vacant positions are reviewed and numbers of males and females appointed to rank-and-file and Section Principals are compared. Career patterns of male and female musicians are also compared. Increases in numbers of women appointed to orchestral posts in the last three decades are compared with increases in the proportion of women in the general workforce. The data of orchestral membership are then compared with the numbers of young people receiving tuition on orchestral instruments retrieved from a large national database (*n* = 391,000 students). Implications for the future of male and female representation in orchestral personnel are then considered.

## Introduction

During the eighteenth and nineteenth centuries, acquisition of musical skills by women was applauded, but social conventions prevailing in Europe and America approved their display in private but not in public. Except for the piano and the voice, women were severely limited in their access to musical training, witness the difficulties suffered by the English composer Ethel Smyth (1858–1944) and by other women (Smyth, 1987; Wood, 1995; Gillett, 2000; Vorachek, 2000; Kertesz and Elizabeth, 2001; Meling, 2016, p. 188). Conventions of respectability and appropriateness regarding feminine manners and appearances and attitudes to the female body decreed that some instruments were “unsightly for women to play, interfering with appreciation of the female face or body” or judged their playing positions to be indecorous (Gillett, 2000; Doubleday, 2008). Female cellists, for example, were obliged to adopt an impractical position sitting alongside the instrument in order to avoid a scandalous indelicacy of placing an instrument between their legs^1^ (Cowling, 1983; Tick, 1986; Doubleday, 2008, p. 18; Baker, 2013).

As a consequence of these social attitudes, women were excluded from professional music-making, and until the second decade of the twentieth century, membership of professional orchestras was restricted to male musicians (Fasang, 2006). The first appointments of women to tenured positions in a major orchestra in the UK were made by Sir Henry Wood, in 1913, by his engagement of six female violinists to the Queen’s Hall Orchestra. The loss of male musicians during the 1914–1918 war brought more women to Henry Wood’s orchestra. By the end of that conflict, their number had risen to 18, but acceptance of women was neither universal nor rapid. Early photographs of major orchestras dating from the 1940s show their membership as resolutely male. Examples from the archives of the London Symphony Orchestra, founded in 1904, show no women until 1942, at which date one lady is visible seated among the 2nd violins^2^^,^^3^.

It was not until 1930 that the first woman was appointed to a tenured fully professional post in an American orchestra, when Edna Phillips joined the Philadelphia Orchestra as its harpist^4^. Ellen Bogoda also made history in 1937 as the first woman brass player to be hired when she was appointed as principal horn player by the Pittsburgh Orchestra (Phelps, 2010, p. 36).

Prior to Wood’s Queen’s Hall appointments, the only opportunities for women to play professionally had been with women-only orchestras, for example, that formed in Berlin in 1898, directed by Mary Wurm, but these were few in number (Collins, 2015). Later examples included the British Women’s Symphony Orchestra, founded 1922 and conducted with energy by Grace Burrows, and the Manhattan-based Orchestrette Classique founded in 1932 by Frederique Petrides. Ironically, the latter orchestra ceased operation during the 1939–1945 World War: as men were drafted into the armed forces, its women members were recruited to the major orchestras that previously had been exclusively masculine.

Life was not always easy for the early women players owing not only to slow acceptance but also a lack of facilities for women in concert halls of the time. Archives of the Cleveland Orchestra include a photograph^5^ of Alice Chalifoux, harpist with the orchestra from 1939, using her harp case as a backstage dressing room^^5^^.

Some orchestras have been markedly slow to admit women to playing positions: the Berlin Philharmonic did not do so until 1982, and the Vienna Philharmonic as late as 1997; prior to that date, women might be engaged regularly (for example, harpist Anna Lelkes) but were not publicly listed as orchestral members or awarded tenured posts.

An international survey of professional orchestras by Allmendinger and Hackman (1995) reported that regional representation of women had increased to 30% (UK), 36% (USA) and 16% (Germany). A later study (Goldin and Rouse, 2000) shows the percentage of women players had increased from approximately 5% in 1940 to 25% by 1990, a development described by Miller (2014) as representing “a sea change in the proportion of women players.” A subsequent review^6^ also dating from 2000 showed the ratio of male to female players in American orchestras had increased to approximately 65% (m): 35% (f)^7^. Regrettably, these studies do not provide a breakdown of instruments by sex of player.

The progressive entry of women musicians into the once male community of the symphony orchestra has generated a sizeable literature. A number of studies have been concerned with sociological effects of these changes on the orchestral community, discussing issues such as inter-player relations, conditions of employment, membership stability, opportunities for personal growth, facilities, and financial resources (Allmendinger and Hackman, 1995). Few studies have provided reliable or comprehensive quantitative data of numbers or discussion of differences in the instruments by played by the respective sexes.

Some published commentary has been more characteristic of journalistic polemic than of evidenced reportage, with headlines such as “Sexism is rife in classical music” (Rhodes, 2014, Guardian Classical Music Blog February 2014); “Orchestras still hostile to women” (BBC News: Entertainment & Arts, 2014); “Women are held back in classical music” (Kennedy, 2014); and “Women in classical music” (Alexander, 2017). Causes of these complaints have been attributed variously to players themselves “… problems brought on by the musicians (who are for the most part precious, egocentric, grandiose and socially stunted),” to orchestras “dominated by male musicians” (Kiek, 2012), to orchestral managers “The gatekeepers’ narcissistic, obtuse, resistant to change …” (Rhodes, 2014), or to music conservatoires and management “… people who run music colleges, chaps who run orchestras” (Kelly, cited Kiek, 2012)^8^.

In the following sections, we present an objective analysis of the current representation of male and female musicians holding tenured positions in orchestras across the world, and the instruments on which they perform.

## Data Collection

### Orchestral Membership

#### Method

**Figure 1:**
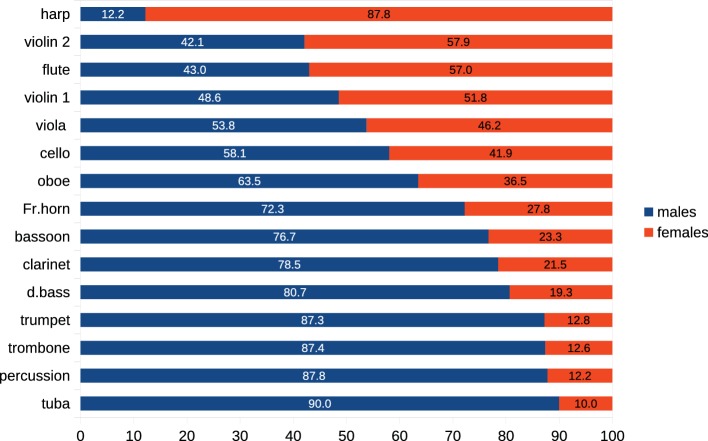
Representation of male and female musicians in 40 orchestras in the USA, the UK, and Europe by instrument and sex of player.

Data of the relative presence of male and female musicians in each orchestral section and for each instrument were collected from the current websites of 40 major orchestras in the UK, North America, and Europe representing a total of 3,420 musicians. Criteria for inclusion in the sample were that an orchestra should be fully professional, recognized as a having world-class status, have made published recordings under established labels, and have accessible data of participant musicians. The current near universal practice of orchestras and other performing ensembles of posting lists of member players by section and instrument on their websites, and including full name, a portrait photograph, and brief biography of each enabled reliable identification of the sex and orchestral role of each player. Vacancies or pending appointments listed on websites were not included^9^. Results of the review are shown in Table 1 and Figure 1.

**Table 1:** Performer populations of 40 orchestras of international standing from the UK, Europe, and the USA by instrument and sex of player.

Instrument	Total *n*	Male (%)	Female (%)	Bias	*p*	Diff. (%)
1st vln	585	48.55	51.75	F	<0.026	3.5
2nd vln	520	42.12	57.88	F	<0.012	15.76
All vlns	1,105	45.25	54.50	F	<0.000	9.00
Viola	422	53.79	46.21	M	<0.012	7.58
Cello	350	58.06	41.94	M	<0.015	16.12
D. bass	254	80.70	19.30	M	<0.000	61.12
Flute	135	43.00	57.00	F	<0.020	14.00
Oboe	148	63.51	36.49	M	<0.001	27.02
Clarinet	149	78.52	21.48	M	<0.000	57.04
Bassoon	147	66.00	34.00	M	<0.000	32.00
Horn	195	72.25	27.75	M	<0.000	44.50
Trumpet	149	87.25	12.75	M	<0.000	74.50
Trombone	127	87.40	12.6	M	<0.000	74.2
Tuba	40	90.00	10.00	M	<0.000	80.00
Tymp/perc.	148	87.80	12.2	M	<0.000	75.60
Harp	40	12.20	87.80	F	<0.000	75.60
**Total**	**3,420**	**60.32**	**39.68**	**M**	**<0.0001**	**20.64**

Modern symphony orchestras list an average of 100–120 musicians; though regional orchestras tend to be smaller than those based in major or capital cities. Overall, across all instruments, a significant majority of the musicians of the orchestras reviewed were male (56.7%, *p* < 0.0001), but there were highly significant regional differences in male-female proportions.

Violin: women were numerically predominant in the violin sections (*p* < 0.000), but assignments of men and women to 1st and 2nd violin positions were not significantly different (*χ*^2^ = 0.73, not sig.).

Flute: A majority of flautists were female (57.04%, *p* < 0.021).

Almost all harpists were female (*p* < 0.0000). A number of orchestras do not list contracted harpists, engaging players whenever repertoire necessitates.

Males predominated in all brass sections (*p* < 0.0001). Where women were present, they were typically found among French horns. In two orchestras, principals in the trombone section were women. In one orchestra, the principal tuba was a woman.

No tympanists were female, and only 12.5% (*p* < 0.0001) of other percussionists were female.

Significant geographical contrasts were evident in the proportions of male and female musicians between those based orchestras in the UK and North America and those in Europe (Table 2). European orchestras typically employed a significantly larger proportion of male than female players, though there were notable exceptions, for example, the Royal Concertgebouw, whose women players constitute just below half the total orchestra’s membership.

**Table 2:** Significance of differences in male/female proportions between orchestra of three regions (March 2019).

Region	Total players	Males (%)	Females (%)
The UK	952	56.0	44.0
North America	1,011	55.9	40.1
Europe	1,903	63.4	36.6
Significance of differences			
The UK vs. Europe	*χ*^2^ = 13.30, df = 1, *p* < 0.000		
The UK vs. North America	*χ*^2^ = 3.06, df = 1, not sig.		
North America vs. Europe	*χ*^2^ = 3.066, df = 1, *p* < 0.008		

**Figure 2:**
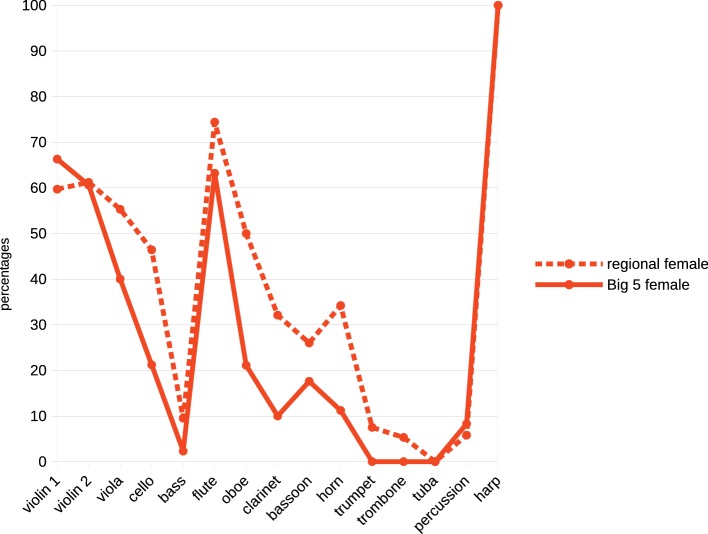
Percentage of women in each orchestral section in “Big 5” and regional orchestras in the USA.

Goldin and Rouse (2000) suggest that more internationally prestigious orchestras have been more resistant to female membership, whereas regional orchestras have included a greater proportion of women. This is consistent with Koskoff’s (1995) earlier report that the proportion of females in an orchestra correlates with its status^10^. To evaluate this proposition representation of women in the so-called USA, “Big 5” orchestras (i.e. New York Philharmonic, Philadelphia, Boston, Cleveland and Chicago orchestras) were compared with that of 25 professional orchestras of other cities in the USA. Results (Table 2) confirmed that less prestigious regional orchestras included significantly more women players than did the orchestras of the “Big 5” (*χ*^2^ = 19.5, df *=* 1, *p* < 0.000), but the increased representation of women was confined to higher-pitched upper strings, woodwind, and harp; brass and percussion positions of all orchestras remained almost exclusively male territory (Figure 2).

When the same test was applied to orchestras in the UK; however, no differences linked to orchestra location were evident: orchestras in the capital did not include a greater proportion of males than did orchestras in the UK provinces (*χ*^2^ = 0.63, not sig.).

### Recruitment Procedures Employed by Orchestras

Recruitment of musicians to orchestral positions is invariably achieved through live audition. Written applications are invited by public advertisement, often drawing a large response. Between 40 and 50 of the applicants are then invited for audition. Typical audition procedure is that candidates are invited to perform at least one set work (usually including the solo part of a concerto), an own-choice work, previously notified extracts from orchestral works, and to read at sight passages from orchestral works. An audition will typically take around 45 min. Juries for auditions normally comprise the Principal and other members of the relevant section (or if the section is numerically large, agreed representatives) plus a representative from related sections of the orchestra. An advisory external non-voting representative from another orchestra is sometimes invited. The orchestra’s principal conductor usually has the right to attend, but this is not often exercised^11^.

It is common practice in orchestras in the USA for the successful candidate to be hired solely on the evidence of the audition: the player judged to be the best performer gets the post. Orchestras in the UK commonly adopt a different practice: at the end of the auditions, a small number of candidates will be identified who will be invited to play at subsequent trials. At each trial, the candidate will attend scheduled rehearsals with the orchestra and then take the vacant chair at the orchestra’s relevant public concerts. This “audition-plus-trials” procedure allows a more comprehensive assessment of a candidate’s sight-reading competence, goodness of fit of tone, and style with that established by the collective orchestra and its director, ability to operate under the stress of a concert and personal adjustment to section members. Its disadvantages are that the sex of the candidate will be known to the jury members. As the selected candidates may each be offered as many as six trials, the process of filling an orchestral chair can become protracted: 2 years would not be exceptional. Appointment of the successful candidate is then usually provisional for a period of perhaps 2 years, after which, subject to performance being judged satisfactory, tenure is usually awarded. Until recently, this “extended trial” procedure has been unique to British orchestras but is now being adopted more widely.

Orchestras do not restrict their considerations to respondents to their advertisements but commonly invite other experienced players whom they have reason to believe may be interested, and these persons are likely to be invited to trials without being subject to audition.

Few instrumentalists achieve positions in elite orchestras end-on to their conservatoire training, though those who are shown to have served for 45–50 years must be presumed to have done so. The majority gain experience in other orchestras from which starting point they later gain promotion, and this is evident from the personal career profiles published on the orchestras’ websites. Smith (1988) evidence from retired musicians suggests that young musicians commonly spend approximately 6 years in less exalted regional ensembles before achieving a chair in a major orchestra.

### Principal Chairs

Each section of a modern orchestra—cellos, flutes, horns, etc.—will have a designated Principal or Section-leader (sometimes referred to as “title chairs”: nomenclature varies locally). Duties associated with these positions are rarely formally documented but are universally understood among professional players. The appointee to each section will be a musician of exceptional performance abilities, with extended experience of the orchestral repertoire and the workings of the orchestral world. They must be competent to advise on stylistic or instrumental issues such as bowing, articulation, and phrasing. The section Principal will normally take any solo passages demanded in the score and will act as agent of the conductor in securing cohesion and unanimity among section members in all aspects of their collective performance (Boersma, 2014; Horvath, 2015). A co/sub-Principal/associate section-leader may also be identified whose role is to assist the section Principal and take over the role and responsibilities when the Principal is not present. Orchestras vary in their practices as to contractual arrangements for Principals. In a numerically large section, such as the violins, it is not unusual for as many as two Principals and two sub-Principals to be listed: in such cases, this usually indicates that contracts with these persons allow for sharing of roles^12^.

“End-of-the-line” players (i.e., those instruments that are nominally included in a section but whose roles are more typically as soloists, for example, piccolo, cor anglais, or bass clarinet) also commonly have Principal status, as do players of single-instrument sections such as tuba, despite the absence of second players of those instruments over whom leadership would be exercised. Management policies differ as to the number of musicians that are identified as Principals within an orchestra, ranging in our sample between a minimum of 12 and a maximum of 29. Some orchestral managements give the appearance of regarding Principal ranking as a reward for long service.

**Figure 3:**
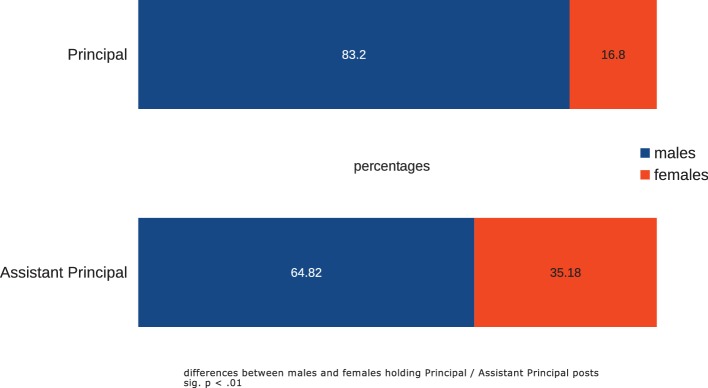
Male and female occupancy of “Principal” and “co/sub/associate-Principal” chairs across 40 orchestras.

Across the 40 orchestras reviewed, 83.2% of persons occupying Principal chairs were male, and 16.8% were women; 64.82% of co-Principals were male, and 35.18% were female (Figure 3) (male-female differences in Principal/co-Principal occupancies significant, *χ*^2^ = 41.29, df *=* 1, *p* < 0.000), but proportions did not differ significantly across the three geographical regions. Some European orchestras, however, have appointed very few women to Principal chairs: the Berlin Philharmonic currently lists only one (2nd Principal viola), Vienna Philharmonic one (Principal harp), the Bavarian Radio Orchestra^13^ none.

### Blind Auditions

At first consideration the evidence of Figures 1, 3 of the unequal distributions of male-to-female representation and appointments as Principals appears to support frequently heard accusations of sex discrimination on the part of selection juries and attitudes of veteran male players, but other associated factors suggest that the issue is more complex. Orchestras do not have target representations of male and female players: the sole aim of the recruitment process is to identity the musician judged best matched to the orchestra’s intended performance product, irrespective of their sex. Nevertheless, the balance of sexes in orchestral populations has frequently been the focus of adverse comment, with frequent accusations of sex-discrimination^14^.

In order to avoid such a possibility, and to counter these repeated allegations, orchestras in the USA have adopted a practice of screening the candidate from the jury so that candidate’s performances can be heard, but their sex is not disclosed. In such situations, screening could fairly be argued to remove any suspicion of sexism. While a few orchestras in the UK also have introduced “blind” auditions, they have not been generally adopted since at trials that follow the audition the sex of the candidates will be evident and screening would therefore not serve its intended purpose^15^.

It has been claimed that increase in numbers of women players reported over recent years is attributable to the introduction of this type of audition procedure (Phelps, 2010; Rice, 2013)^16^^,^^17^. Such claims could be valid only for orchestras where blind audition has been fully adopted and may therefore be treated with caution.

### Length of Engagement

**Figure 4:**
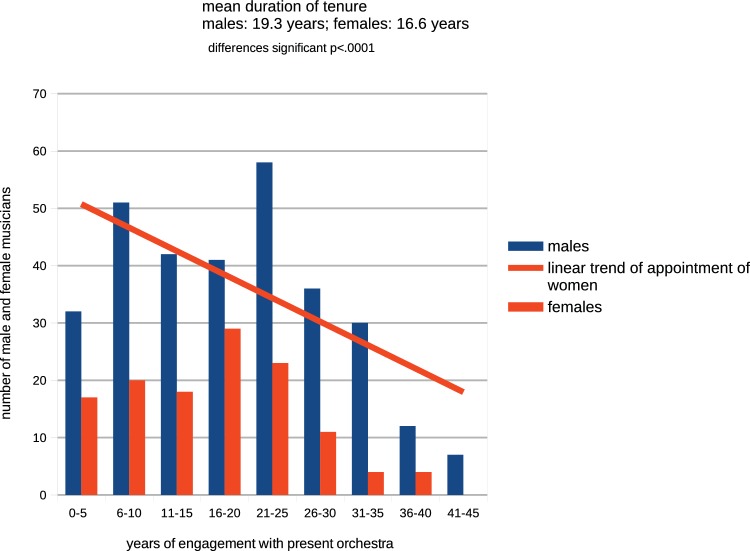
Years of engagement by male and female musicians of four orchestras.

Four of the 40 orchestras reviewed (Cleveland, London Symphony, Royal Concertgebouw orchestras, and Berlin Philharmonic) include the dates of appointment of their musicians to the orchestra in the website biographies. From analysis of these, it is possible to map the typical career patterns of their collective 403 member musicians. Figure 4 shows that current engagements with orchestras are characteristically of considerable length, with a mean duration of 18.5 years. Men typically serve more years than women (mean duration for men of 19.3 years compared with 16.65 years for women, differences significant *t* = 6.26, df *=* 9, *p* < 0.0001). Twelve male players are shown to have served between 40 and 51 years, suggesting that musicians do not lose their skills early, but only one long-serviced woman is reported to have spent as long as 40 years with her orchestra. These extended career patterns are consonant with those reported from Smith’s (1988) interviews with 14 male musicians retired from major orchestras in the USA whose ages at point of retirement had ranged between 47 and 78 years (mean 66.4 years) and whose performing careers had ranged between 23 and 57 years in length.

The mean length of service of Principals and assistant/co-Principals with present orchestra was 17.15 compared with the mean duration for all players of 17.5 years.

Current ages of members of the four orchestras are not published on their respective websites, but Gembris and Heye (2014) provide indicative generic information from 2,536 musicians from Germany’s 132 publically funded orchestras (*Kulturorchestern*), ranging from local to metropolitan, reporting an age range of 20–69 years with a mean age of 45.25 years.

It is evident from Figure 4 that career patterns of male and female instrumentalists differ. The point of maximum representation of women instrumentalists is at approximately 16–20 years of service, after which their numbers progressively decline. Men reach their maximum representation later at around 20–25 years’ engagement, after that point their numbers also decline but over a more extended period than women. Two important consequences result from this contrast: firstly, the average age of women players in an orchestra is lower than that of its men^18^, and secondly that even if all appointments of new players to vacant positions were to be equally distributed between the sexes, because of the more extended length of service of males, logistically an orchestra would include a greater number of men than women.

Because of these typically extended lengths of tenure of both sexes, few positions become vacant in an orchestra any year: from Figure 4, it is evident that only 49 appointments have been made across all four orchestras during the most recent five-year period.

### Women’s Presence in Orchestras in Relation to General Labor Market

**Figure 5:**
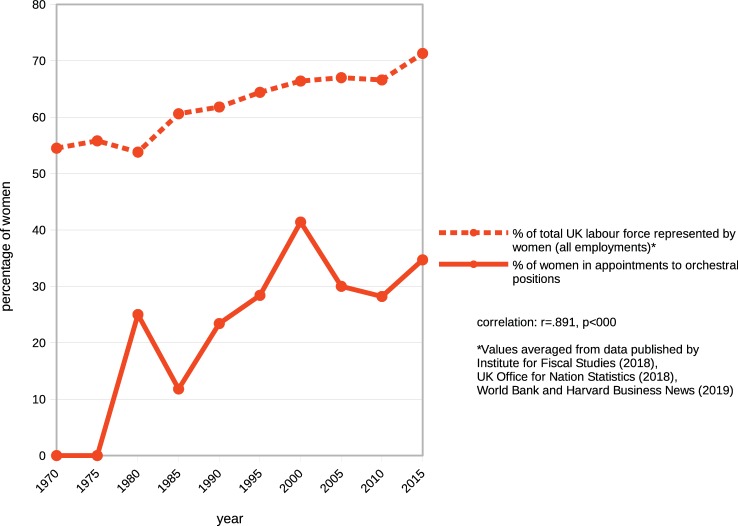
Growth trends in women’s orchestral membership during the period of 1970–2015 compared with growth trends in women’s presence in the overall workforce.

It is evident from the linear trend at Figure 4, that, over time women have been gaining an increasing presence in symphony orchestras. Using statistical reports from official sources, this trend can be mapped against a parallel increase in women in the general labor force successive 5-year periods (Figure 5).

Mascherini et al. (2016) report that the increase in women’s participation in the workforce has been the most significant change in labor markets since 1950, reflecting universal changes in social attitudes to the employment of women^19^. Allmendinger and Hackman (1995) propose that this is an underlying factor in the increasing presence of women in orchestras. The high correlation between the two plots of Figure 5 confirms that orchestral appointments have fully reflected the increased presence of women in the general workforce.

### Relation of Pre-conservatoire Students in Tuition to Representation of Sexes in Orchestras

Over the past 30 years, an extensive literature has accrued reporting differential associations of musical instruments, some instruments perceived to have feminine connections, others masculine. Judgments of these associations have proved to show high reliability (Stronsick et al., 2017). This has given rise to a social perception of sex-appropriateness of instruments, a notion that appears to be widely acknowledged, and held equally by both sexes (O’Neill and Boulton, 1996). This dimorphism has frequently been characterized as due to a form of sex stereotyping.

Despite the considerable number of related studies and commentaries, a definitive ordering of perceived gendering of instruments—most “feminine” to most “masculine”—has been difficult to determine owing to inconsistency among researches in the instruments that have been considered, differences in the musical genres with which they have been associated, and in the target populations addressed. A study by Hallam et al. (2008), however, reports substantial data of children receiving instrumental tuition in a large geographical area of the school system in England. Their report provides numbers, sex, and instruments studied from a substantial cohort of over 391,000 students within the age range of 5–18 years, covering instruments associated with a broad range of musical genres. Assuming that few of the children would be learning musical instruments against their own wishes and within possible limitations of availability of tuition and instruments, the data published by Hallam et al. may be regarded as a reliable indicator of the instruments that children elect to learn and enjoy playing (Rife et al., 2001).

**Figure 6:**
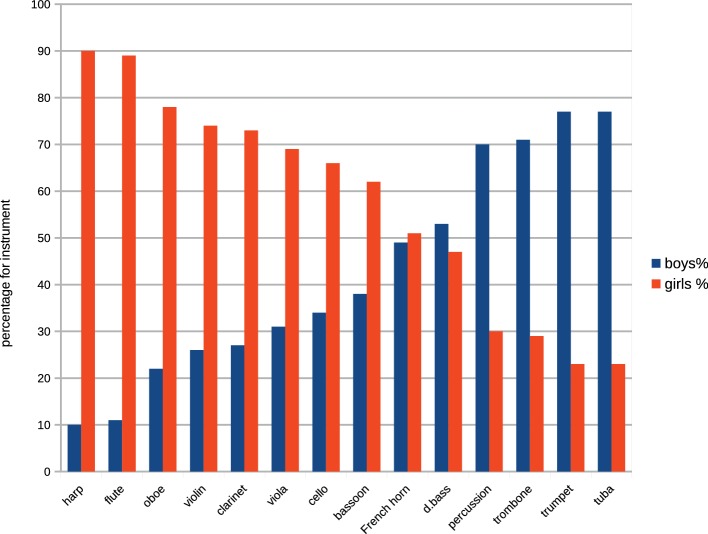
Percentages of girls and boys receiving tuition on each orchestral instrument.

By comparing the numbers of male and female students receiving tuition on each of the orchestral instruments listed in the Hallam et al.’s study, a binomial probability could be calculated for the gender distribution of students for each instrument, and by ranking the probability levels according to magnitude, an order of gendering could be determined. Although the numbers of students relating to individual instruments inevitably showed wide variance (for example, harp *n* = 58; violin *n* = 75,763), no instruments had a total *n* that was sufficiently small for their associated probability value to be considered unreliable. Instruments for which the number of girl students significantly exceeded the number of boys were listed as feminine type, those for which the number of boys significantly exceed girls were listed as masculine type. Instruments for which the boy-girl binomial probability proved to be statistically not significant were considered to be gender-neutral. From these values, it was possible to construct an hierarchy of instruments by sex type, most feminine to most masculine (Table 3). Because of the large variance of *N* (i.e., numbers of students studying the various instruments), the order is expressed as percentages at Figure 6.

**Table 3:** Order of sex typing of orchestral instruments based on proportions of girls and boys receiving instrumental tuition on each instrument (ordered from data of Hallam et al., 2008).

**Feminine-type**		
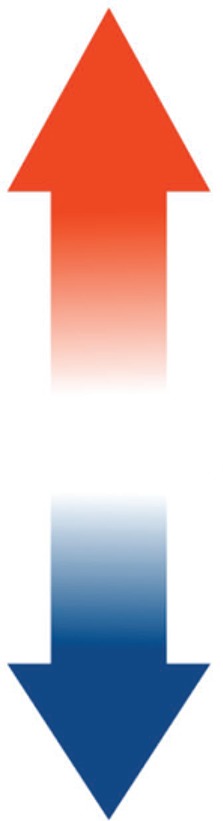	Harp Flute Oboe Violin Clarinet Viola Cello Bassoon	
	French horn Double bass Percussion Trombone Trumpet Tuba	gender-neutral gender-neutral
**Masculine-type**		

The resulting order above accords well with an evidence of studies that have investigated variously: attitudes among pre-service and in-service teachers (Abeles and Porter, 1978; Griswold and Chroback, 1981; Tarnowski, 1993; Zervoudakes and Tanur, 1994; Bayley, 2000; Yoder and Kahn, 2003; Johnson and Stewart, 2005; Abeles, 2009), gender stereotyping (Crowther and Durkin, 1982; Lipton, 1987; Byo, 1991; Bruce and Kemp, 1993; Elliot and Yoder-White, 1997; Pickering and Repacholi, 2001; McKeage, 2004; Walker, 2004), and the perceptions of children (O’Neill and Boulton, 1996; O’Neill, 1997; Ryan et al., 2000; De Vous, 2011).

The median point of the pitch compass of each instrument was calculated by reference to standard texts on orchestral instruments. The correlation between the order of pitch height at Table 3 and sex-type order was highly significant at *rho* = 0.720, *p* < 0.006, demonstrating a strong connection between popularity of instruments, pitch height of their sounding ranges, and sex of students.

By comparing the Hallam et al.’s dataset with our data for male-female orchestral representation above, it was also possible to measure the level of correspondence between the numbers of male and female students receiving tuition on each instrument with the numbers of professional artists in post in the orchestras sampled. The levels of correlation were high and highly significant for both sexes. Despite these high correlations, however, the analysis revealed a marked and consistent discrepancy between the proportions of male and female students who achieved orchestral positions. The comparisons at Figure 6 show that with the exception of two woodwind instruments, fewer women achieve orchestral positions than would be predicted by the numbers of girls receiving instruction (*t* = 2.96, df *=* 26, *p* = 0.006), and conversely, a greater percentage of men achieve orchestral positions than would be predicted by numbers of boys receiving tuition (*t* = 2.96, df *=* 26. *p* = 0.006).

It is evident from the plots for both the students and the in-post professionals that both groups adhere to characteristic sex-typed instrument choices illustrated in Table 3. The reasons underlying these sex-dimorphic choices have been the subject of extensive research, and a number of factors have been proposed as possible causes of instrument gendering of which the most frequently discussed in the literature are:

1. Preference for quality of sound/timbre (Griswold and Chroback, 1981; Delzell and Leppla, 1992; Fortney et al., 1993; Elliot and Yoder-White, 1997; Graham, 2005; Chang, 2007; Varnado, 2013; Payne, 2019);
2. appearance of an instrument; its manner of playing (Burr, 2003);
3. approximation of pitch range of instruments to player’s vocal range (Sinsel et al., 1997; De Vous, 2011; Nannen, 2017; O’Bannon, 2017);
4. personality factors (Builione and Lipton, 1983; Sinsel et al., 1997; Katzenmoyer, 2003; Chang, 2007; Hallam et al., 2008; Abeles, 2009; Payne, 2019);
5. educational and training opportunities, attitudes of teachers and music directors, and lack of female role models in secondary and higher education (Bayley, 2000; Graham, 2005; Johnson and Stewart, 2005; Gould, 2008; Gathen, 2014);
6. socio-economic factors (Katzenmoyer, 2003; De Vous, 2011; Varnado, 2013);
7. gender stereotyping (Byo, 1991; Bruce and Kemp, 1993; Elliot and Yoder-White, 1997; O’Neill, 1997; Pickering and Repacholi, 2001; McKeage, 2004; Walker, 2004; Hallam et al., 2008; Abeles, 2009; Benet Crowe, 2010; Varnado, 2013; Wrape et al., 2016; and numerous others).

### Instrument Choice

A prominent feature of young students’ choice of instruments that has received considerable attention is the characteristic preference by girls for higher-pitched, smaller and lighter instruments, and boys for lower-pitched, larger, and heavier instruments (Hallam et al., 2008; De Vous, 2011), and this phenomenon is sharply evidenced in Figure 6.

In a rarely cited study, De Vous (2011) asked 4th and 5th grade band students within a school district to state the reason for their choice of instrument. Analysis of the widely varying responses shows that some attributed causes featured consistently: over 30% mentioned social and gender pressures (It is a girls’ instrument/I think it is for girls/ boys do not play it/there is only one boy in the flute class/more likely to be a guy instrument/mostly boys play it, etc.), 19% preference for tone quality of the instrument (it is a delicate sound/it is peaceful/boys have louder instruments/it sounds boyish), and 11% the pitch of their chosen instrument and its relation to own voice range (it has a high sound/it is a manly sound/because boys have darker voices/they have lower voices/it sounds like a boy would), and these last forms of response especially came from boys.

To test the validity and reliability of these two postulated associations, the median point of the pitch compass of each instrument listed in the sex-type order table (Table 3) was calculated from standard texts on instrumentation^20^. The ranked median values were then compared with the sex-type rankings. Results showed a highly significant association between sex typing of instruments and their sounding pitch ranges, *rho* = 0.730, *p* < 0.000, *tau* = 0.581, *p* < 0.003, confirming the proposition that females show preference for higher pitched instrument and males for those of lower pitches.

Similarly, instrument weights were ascertained^21^ by direct measurement of sample instruments, from websites of instrument manufacturers and dealers, catalogues of major dealers (e.g., Thomann GmbH; Besson London, etc.) and relevant publications (e.g., Sirr and Waddle, 1997; Waddle and Loen, 2003; Waddle et al., 2003). The order of instruments by weight^22^ was then compared with the sex-type order of Table 1. A highly significant association was observed (*rho* = 0.841, *p* < 0.001; Kendall’s *tau* = 0.692, *p* < 0.001), confirming the proposition of Hallam et al. and others that females show a preference for smaller, lighter instruments, whereas heavier and larger instruments are preferred by boys.

Correlations carry no implications of causality, but these results indicate that the sounding pitch, size, and weight of instruments are factors significantly associated with instrument choices of both sexes.

### Review, Commentary, and Questions for Further Research

The comparative representation of men and women in professional orchestras is an issue of considerable complexity in which many disparate variables intersect. On *prima facie* evidence, our review of 40 world-class orchestras shows that overall male musicians have a greater numerical presence, significantly outnumbering females by a ratio of 60% males: 40% females. Within these simple proportions of imbalance, however, other significant contrasts are present: between orchestras of different geographical regions and their contextual employment and promotion structures, between individual orchestras, the respective career patterns of male and female musicians, their ages and lengths of tenure of positions, and the instruments they play.

#### Regional Differences in Sex Representation

Our review show that balance of sexes in orchestras varies significantly according to region. Small differences in the presence of women between those in the UK (44%) and the USA (40%) were not statistically significant, but orchestras in Europe showed a significantly lower female presence (36%, *p* < 0.0001), though there were some notable exceptions.

Manturzewska (1990) observes that professional life and development of musicians are strongly influenced by socio-cultural factors. Prior to the 1930s, historic work roles of men and women went largely unchallenged: men went to work daily and earned the family’s income, women worked at home maintaining good household order and fulfilling maternal obligations and needs. During the period of World War II, the need for women to take on work roles previously assigned exclusively to males brought about profound revision of general public perceptions of gender-appropriateness. As a consequence, work roles of men and women have increasingly become convergent (Norman et al., 2017). Women now take leading roles in all aspects of science and technology, medicine, finance, business management, government, law, aerospace, universities, and religious ministry and are appointed to senior ranks of command in the police and armed forces.

**Figure 7:**
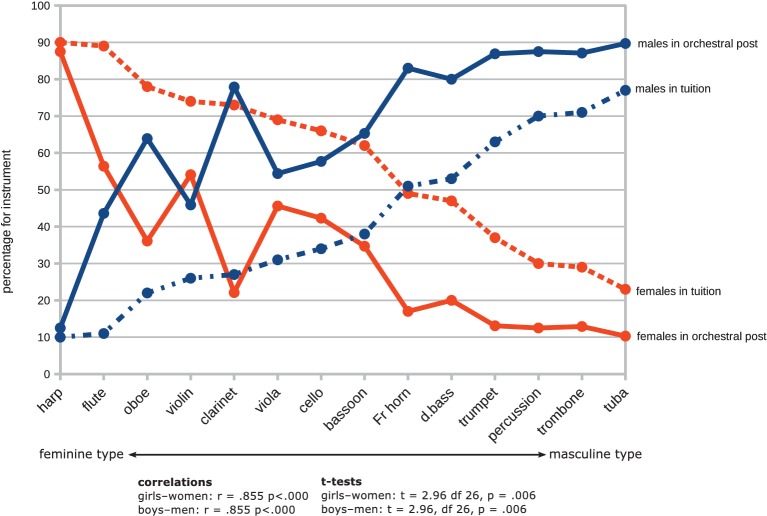
Relationship between student instrumentalists (data from Hallam et al., 2008) and musicians in orchestral post.

In view of these changes in societal attitudes toward women in employment, and because in many countries differentiation in employment on grounds of sex is now unlawful, it could be expected that men and women would have an equal presence in every walk of life. Changes in public attitudes and actual life roles do not take place rapidly, however; rather they adjust over extended periods of time (Figure 7). It is only after public perceptions have reached a point of near universality that governments are moved to institutionalize them in legislation. But legislation is prospective, affirming intentions for the future, and cannot apply retrospectively. Existing obligations in employments will remain operative until the expiry of their terms, and orchestral musicians will remain with their orchestras until their elected time of their retirement. Only at that point will a chair become vacant. Adjustments to the overall balance of sexes in orchestras can therefore only take effect over a relatively extended period of time.

#### Contextual Social Attitudes

**Figure 8:**
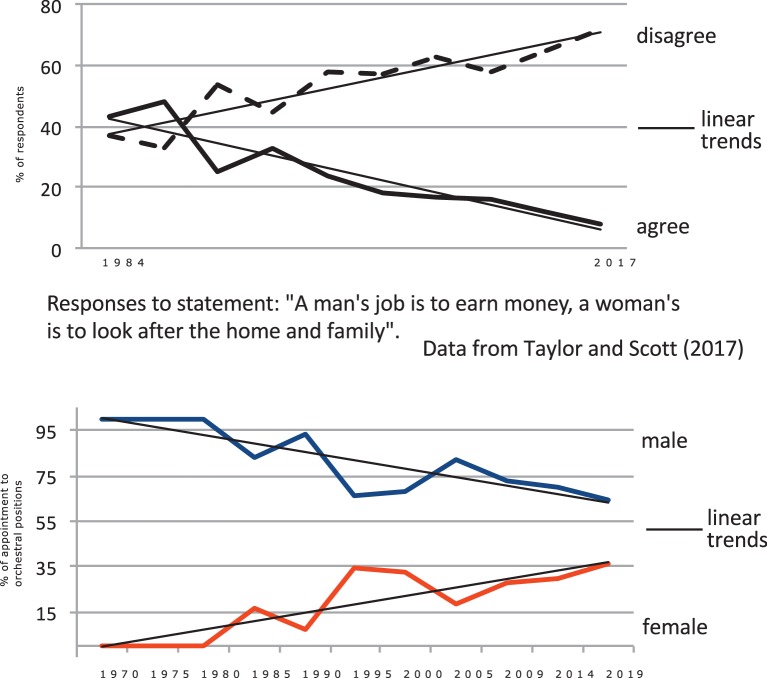
Comparison: change in societal attitudes to gender roles with appointment to orchestral positions of males and females.

Juxtaposition of graphs in Figure 8, below, illustrates how changes in societal attitudes to women’s employment outside the home and their entry into the general labor force over 30 years have been reflected in increased appointments of women to orchestral positions period (Alwin et al., 1992; Taylor and Scott, 2018; OECD, 2019).

**Figure 9:**
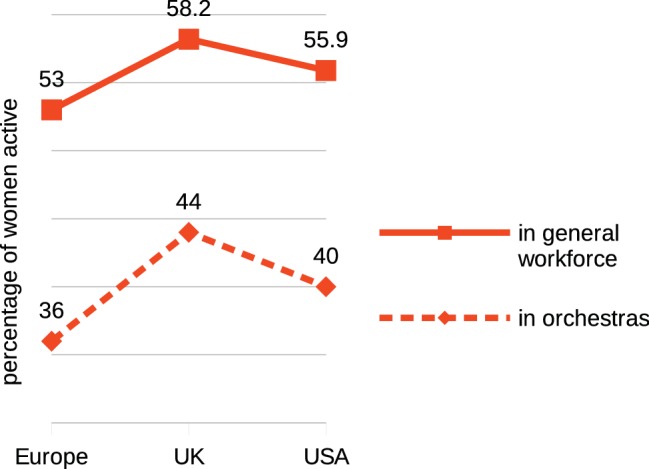
Comparative representation of women in orchestras and women in general labor force by regions 2018.

Correlation between responses “disagree”^23^ (Figure 8, top) and increases in appointments of women to orchestral posts (Figure 8, bottom) is highly significant at *r* = −0.90, *p* < 0.000. It is evident that representation of women in orchestras has increased linearly over the past 50 years, and the rate of increase has been fully commensurate with changes in social attitudes to women’s employment and the consequent growth in women’s engagement in the general labor force (Figure 5). A further analysis of this relationship (Figure 9) across the three regions shows that the region with the lowest representation of women in orchestras (Europe) is also the region with the lowest presence of women in the labor force and conversely the region with the highest orchestral presence (UK) has the highest level of women in the labor force, strongly supporting Manturzewska’s (1990) assertion.

#### Effects of Ages of Retirement

Mandatory ages of retirement are now unlawful in many Western societies: so long as employees are judged to be fully meeting the operative standards of their employment, they will remain in post. These changing contextual societal attitudes may be welcomed on grounds of anti-ageism, but since our data show that women leave their orchestras earlier than do males, this will necessarily affect the balance of sex representation in orchestras. It may also bring an increase in numbers of older male players remaining in post for sustained periods.

#### Section Principals

Overall, mean length of service of Principals and sub/co-Principals with their present orchestras was 17.15 years, marginally but not significantly lower than the mean for all players at 17.5 years. Suggestions that orchestras typically treat appointment as a section Principal as a long-service reward are therefore not supported overall by our evidence. Nevertheless, there were marked differences between orchestras in the ages at which principals were appointed, and highly significant differences in their sex balance: male principals outnumbered females by a ratio of 65%:35% and these unequal proportions in positions were characteristic of orchestras of all three regions. Anecdotal evidence suggests that it is often female members of a section who are resistant to appointment of a woman as their sectional principal (Aboud, 2010). The underlying reasons for this situation could only be determined from observation of appointment and audition procedures while they are in progress and from discussions with orchestral managers.

#### Context and Location of Orchestras

There is clear evidence that in the USA, the proportion of women musicians varies with the status of the orchestra: those counted as constituting the “Big Five” include a significantly smaller proportion of women than those in provincial cities. The UK orchestras did not conform to this pattern: proportions of women in orchestras located in London did not differ from those in regional locations. A possible explanation of this is that the British Broadcasting Corporation organization maintains orchestras in both regional locations and in the capital, and working conditions and remunerations in all five are subject to identical agreements between BBC and the Musicians Union of the UK (BBC/MU Orchestras agreement 2014-17, CBSO, 2017).

#### Sex Differences in Choices of Instruments

Sex differences in choices of instruments are clearly evident in the Hallam et al.’s data (graphed at Figure 7). In a following study (Hallam et al., 2017) researched the practicing habits of 3,252 boys and girls in the UK through a self-report questionnaire; no statistically significant sex differences in weekly practice time or motivation to practise were found, though girls used more systematic practice strategies and applied more immediate corrections, and boys showed higher levels of concentration. There are therefore no grounds to anticipate that boys might be more successful in their studies than girls or would be more successful in gaining admission to conservatoires. Neither do statistics of admissions to conservatoires reflect such difference.

**Figure 10:**
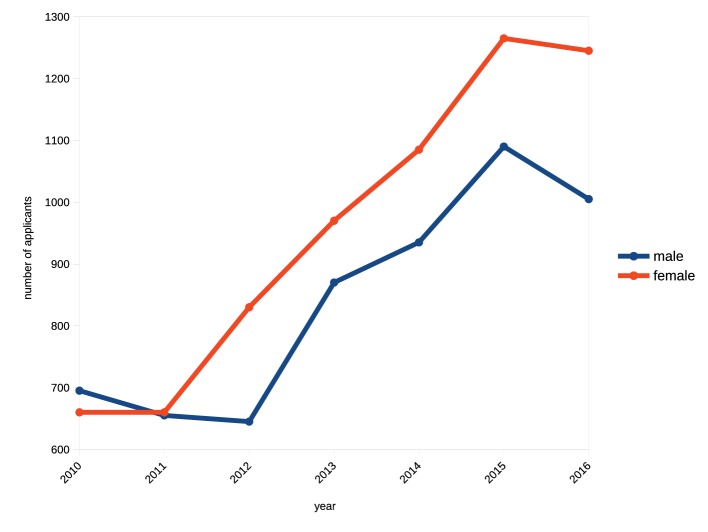
Acceptances of applicants for admission to the UK Music Conservatoires 2010–2016.

Is there any merit therefore in Kelly’s (cited Kiek, 2012) contention, above, that music conservatoires are in some way responsible for unbalanced representation of sexes in orchestras? Analysis of admission records published in successive annual reports of UCAS Music Conservatoires shows that of the 8,200 applications for admission to six UK state conservatoires^24^^,^^25^ across the 7 years 2010–2016, applications from women candidates exceeded those from men by approximately 20%. Offers of student places across the same period show similar proportions (Figure 10): women received more acceptances for admission in each year than did men. There is no evidence in these data of bias toward admission of males rather than females to advanced courses of music training, and there is no statistical justification for Kelly’s comments.

**Figure 11:**
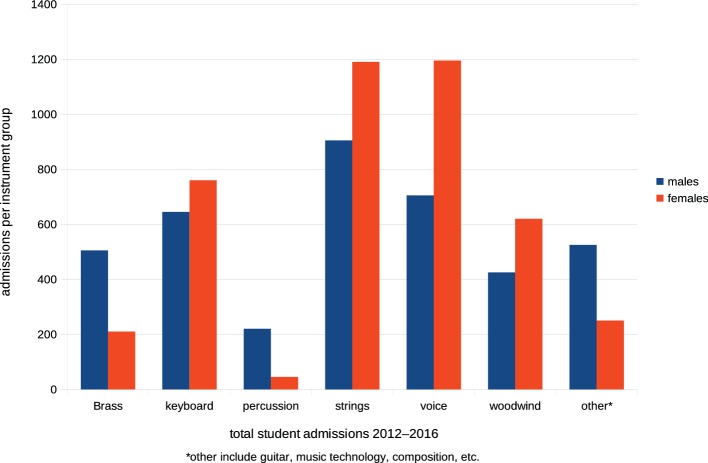
Admissions to the UK Music Conservatoires over a 5-year period of 2012–2016 by instruments.

As could be expected, distribution of instruments played by male and female conservatoire applicants is closely conformant with that of younger students in tuition at Table 2, with female entrants preferring to study strings and woodwind and males showing preferences for brass and percussion (Figure 11).

**Figure 12:**
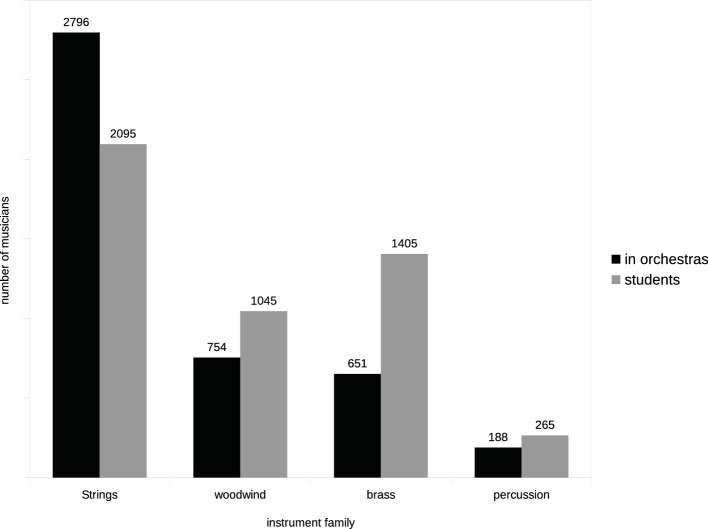
Comparison of conservatoire student instrument choice with needs of orchestras.

The above distributions also accord well with the proportion of musicians playing those instruments in orchestras (Figure 12), though some values suggest that if these patterns were to continue as at present a shortfall of players in string sections is likely in the future.

Despite this satisfactory growth in the presence of women, some marked anomalies in male-female representation remain. Our review has shown that fewer women achieve orchestral positions than would be predicted by the numbers of girls receiving instruction and conversely, a greater percentage of men achieve orchestral positions than would be predicted by numbers of boys receiving tuition and the proportion of male conservatoire students. Given the absence of sex bias in the entry data of conservatoire admissions above, identifying causes of this disbalance will require investigation of career intentions and life-style expectations of both male and female students, especially at the stage of completion of conservatoire training.

#### The Future Outlook of Sex Representation in Orchestras

Our data shows the disbalance of male and female musicians in orchestras to have two dimensions: (1) unequal overall representation of men and women and (2) skewed distribution of males and females across instrumental sections associated with the proclivity of women for smaller, lighter instruments of higher pitch, and men for larger, heavier, instruments of lower pitch, as prominently evidenced in the data of Figures 1, 7.

Reasons for the numerical disbalance are primarily historical, having their origins in a male-dominated labor force of post-Victorian society and its pervasive influence on attitudes to the respective social roles of men and women, especially those concerning child bearing and family responsibilities. The intensive work schedules of an orchestra are not easily accommodated with family life, though there are many examples to show that it is not impossible. Kiek (2012) argues that orchestral managements should improve their provision for family life, pointing to the model of Scandinavia where she claims “rehearsals take place during the school day, tours are never in school holidays, and part-time jobs are on offer.” Orchestras are expensive organizations with revenue needs that bring a reliance on concert engagements away from the home base concert hall, often involving periods in distant continents. Kiek’s examples of working conditions and arrangements in orchestras would not be recognized by the majority of professional musicians.

#### Sex Typing of Instruments

The conspicuous discrepancy between the proportions of male-versus-female students receiving instruction on orchestral instruments and the representation of their respective sexes in orchestral membership raises complex questions. It is beyond the scope or purpose of the present paper to give them adequate consideration, but it is probable that the causes are associated with sociological and psychological issues such as personality, self-imagery, career expectancies, and choices, preferred lifestyles and education.

#### Instrument Choice

What factors influence young people when they choose the instrument they will learn? Reasons are likely to be complex, involving both socio-cultural and practical issues, and therefore difficult to identify with accuracy. Apart from the small-scale investigation by De Vous (2011), there appears to have been relatively little address to this question. Until the relevant factors are clarified, commentaries blaming parents and teachers for following prevailing stereotyping remain at a level of conjecture. Large-sample investigation with young students soon after their point of commencement of instruction, simultaneously examining availability of instruments, appropriate teaching, and financial and socio-cultural issues is needed to bring clarification.

#### Effectiveness of Blind Auditions

Since their first introduction in the 1970s, blind auditions for orchestral appointments have generated much commentary particularly in the press. The impression is given that they have now been adopted universally and have brought about a revolution in the male-to-female representations and rid the orchestral world of its supposedly endemic sexism. The headline of Rice’s feature of 2013 “How blind auditions help orchestras to eliminate gender bias” is one of the more moderate contributions.

#### The Totality of an Orchestra

The phenomenon we call music begins with the composer who assembles sounds to create sequences of musical gestures that enact his inner musical and emotional experiences: these are encoded in notational form in the musical score. The information encoded in the score sets out only the material structures of the composer’s ideas: it cannot encapsulate the emotional experiences that engendered the composition—its “feelingfulness”—nor encode quality of tone, rhythmic, or dynamic articulation or other nuances of performance. These qualities are realized from the score by the performer. The sounds perceived by a listener are therefore a synthesis of the sounds first envisioned in the composer’s imagination overlaid by new qualities that are products musical imaginings, emotions, and experiences of the performer. In the case of an orchestral performance, this new realization depends on the powers of re-envisioning of the musical director engaging the artists of the orchestra in a collective re-enactment. This is achieved through communication in rehearsal sessions and by the subtlety of the director’s gesture and the collective artistry of the orchestra’s musicians. An orchestra is therefore a dynamic unit whose purpose is to realize the ideas of the composer in actualities of sound, re-imagined through the orchestral director, and realized in sonic acts by the orchestral players.

Unanimity of style and intention in this endeavor are the quintessential qualities of a fine orchestral ensemble. Technical competencies at the instrument are only basic necessities expected of a musician at audition. Compatibility of sound production with developed qualities of the ensemble: quality, attack and release of tone, extent and rate of vibrato, articulation of bowing, awareness and sensitivity to subtleties nuance empathy to nuance, and rhythmic articulation are among critical qualities. Principal players are soloists in their own right and their performance qualities and style are critical to an orchestra’s stylistic personality. It is therefore not surprising that orchestras exercise considerable care in their recruitment procedures in their efforts to secure appropriate appointees.

Advertisements for positions in major orchestras will commonly draw around a hundred applications: this number will be reduced at the paper level to some 50 or 60 who will be invited for audition. From this large pool, five or six will be selected for trials. Sometimes, after the trials have taken place, none of those selected are found to be satisfactorily matched to the orchestral ensemble, and the advertisement and application process may be repeated, sometimes more than once. Additionally selection panels do not regard themselves as restricted to those musicians who respond to the advertisements but may invite players in post with other orchestras to apply. The appointment process may therefore take many months before a universally approved player is found.

From the forgoing, it will be understood that calls, such as Kiek’s (2012) for legislation requiring predetermined proportions of males and females in orchestral personnel, apart from compromising hard-won legislation against sex discrimination in advertising and recruitment in both the USA, the UK, and Europe, could lead to inability to appoint a most-favored candidate, with consequent deleterious effect on quality of artistic ensemble.

Engagements with world-class orchestras are typically long-tenured and owing to the tendency to late retirement ages, especially of male musicians, vacancies for new recruits occur relatively infrequently—an example of unintended consequences of legislations. This has caused change in the balance of sexes in the personnel of orchestras to take place rather slowly. Nevertheless, many orchestras are approaching numerical equality: British orchestras have already achieved that state, and those of North America are close to it. A number of those in Europe are still some distance away, however. If screened auditions, where they are appropriate, do protect women from possible bias at auditions, equality may be achieved, but if women continue to favor higher pitched instruments, given the number of brass and lower string players in the composition of a symphony orchestra, overall equality may prove elusive.

## Data Availability

The datasets generated for this study are available on request to the corresponding author.

## Author Contributions

DS and EH claim authorship of this manuscript and also the copyright of the research work that this manuscript reports.

### Conflict of Interest Statement

The authors declare that the research was conducted in the absence of any commercial or financial relationships that could be construed as a potential conflict of interest.

## Footnotes

^1^As illustrated in “The home quartet: Mrs. Vernon Lushington and her children” painting by Arthur Hughes, (1882–1883); also “Concert champêtre” by Bonifazio de’Pitati (1487–1553); “A Musician Playing the Violoncello in a Landscape with Classical Statuary and Ruins” by Jan Jozef Horemans (1682–1759). The difficulty of this position was exacerbated by the lack of an endpin to support the body of the instrument, common practice in earlier times (Braun, 2015).

^2^https://lso.co.uk/orchestra/history/chronology-alt/1900s

^3^https://cso.org/about/rosenthal-archives/

^4^Not all players in earlier orchestras were fully professional, for example, the Principal Bassoon of the Toronto Symphony, Dr. George MacRitchie, was primarily known as a dentist.

^5^https://www.clevelandorchestra.com/from-the-archives/from-the-archives/2016-04-15-harp/

^6^https://classicfm.com//discover/latest/gender-inequality

^7^https://www.classicfm.com/discover-music/latest/gender-inequality-american-orchestras/

^8^https://www.bbc.co.uk.news/entertainment-arts-25581668

^9^Orchestras vary in the ways that their players are listed: some list only the core body of their contracted players and do not list “extras” (players who do not have permanent contracts but are called when a program demands large orchestral forces), and others list all associated players. This may give a false impression that some orchestras are much larger than others. Anecdotal information from players suggests that “extras” are more often female than male.

^10^Goldin and Rouse (2000) also report that before 1980 none of the “Big 5” USA orchestras (i.e., New York, Boston, Chicago, Cleveland, and Philadelphia) had more than 12% women membership.

^11^The jury for auditions for European orchestras has commonly comprised the entire orchestra (see report of James Galway’s audition with Berlin Philharmonic. Ramey (2015), at http://flutecorner.rameymusic.com/galway.htm). This must have been a daunting experience for candidates. Recent advertisements for vacant posts in these orchestras suggest that jury numbers have now been moderated. “A View from Germany: Classical Music is so Sexist – Women’s Philharmonic Advocacy.” Accessed April 8, 2019. https://wophil.org/a-view-from-germany-classical-music-is-so-sexist/

^12^As Hackman has observed (Judy, 1996), role sharing is an important aspect of orchestral employment for highly skilled musicians: it recognizes their personal status as eminent players and artists capable of careers as soloists in their own right, allowing a musician to develop a supplementary career as a soloist and therefore encourages recruitment of world-class players.

^13^BRSO, Symphonieorchester des Bayerischen Rundfunks.

^14^“Alarming gender inequality” (Classical FM/discover music/); “Gender bias in international orchestras” (J. Osborne, IAWM journal 1996); “Fighting the gender inequality in classical music” (S. MacRae, The Scotsman, 14.08.2017); “Sexism is rife in classical music” (J. Rodes, Classical Music Blog, February 4, 2014); “Classical music is so sexist” (I. Kloepfer, Frankfurter Allgemeine Zeitung, 13.08.2018); and “Orchestras can be a hostile place for women” (H. Singh, Daily Telegraph, 24.01.2014).

^15^Blind auditions can be helpful to both a candidate and a jury member where the former may be a student of the latter.

^16^There still may be problems in that screens do not necessarily reach full to the ground. Koskoff (1995) cites cases of women candidates reporting having worn heavy shoes at an audition and walked with a male stride lest, their sex is detectable below the screen (Goldin and Rouse, 2000). In cognizance of this problem, candidates have sometimes been invited to remove their shoes before moving behind the screen.

^17^In Germany, retirement age is currently 67, but this is under revision. No compulsory ages of retirement are operative in the UK or the USA, and musicians may therefore serve until they wish to retire. It is therefore possible for veteran musicians to continue working to advanced ages with the proviso that should their performance standards be perceived as failing they may be asked to retire, or required to undergo an appraisal audition.

^18^Professional musicians suffer high rates of musculoskeletal pain. Of a sample of 243 elite musicians, 86% reported experiencing regional pain in the past 12 months affecting neck, low back, and shoulders, varying with instrument played. Women are shown to be at higher risk than men, which is a possible factor in their earlier departures from orchestral employment (Leaver et al., 2011).

^19^See also Thévenon (2009), Roantree and Vira (2018), OECD (2019), Powell (2019), etc.

^20^Harmonics and notes achieved addition of crooks or other mechanical means of pitch range extension were disregarded.

^21^There is no satisfactory way that the volumes of instruments could be compared, but since volume may be considered as relative to weight, the latter is taken as a valid measure for both dimensions.

^22^The harp, though a strongly gendered instrument, was excluded on grounds that its non-portability rendered it irrelevant to this exercise.

^23^That is, rejecting previous attitudes.

^24^Covering Birmingham Conservatoire, Royal Academy of Music, Royal College of Music, Royal Northern College of Music, Royal Conservatoire of Scotland, Royal Welsh College of Music and Drama, and Trinity Laban Conservatoire of Music and Dance.

^25^Owing to the diversity of training institutions in the USA and Europe, there is no comparable collective source of information of admissions for these regions. UCAS is the UK Government administrative body responsible for oversight of entry to courses of higher education.
